# EXPLORING THE PHENOMENON OF BOREDOM IN THE CONTEXT OF LEISURE AMONG OLDER ADULTS

**DOI:** 10.1093/geroni/igad104.2724

**Published:** 2023-12-21

**Authors:** Jaesung An, Laura Payne, Megan Janke

**Affiliations:** California State University East Bay, Hayward, California, United States; University of Illinois Urbana-Champaign, Champaign, Illinois, United States; Berry College, Mt Berry, Georgia, United States

## Abstract

Boredom is a concept that seems at odds with leisure, yet research on this topic indicates these constructs are often connected. Leisure boredom has been studied primarily among adolescents (Donati et al., 2019; Weybright et al., 2015). However, boredom is prevalent among older adults. Social isolation and loneliness often intertwine with boredom, and research suggests that prolonged boredom can negatively affect health and well-being (An et al, 2022; Goldberg et al., 2011). Yet, there is a dearth of research on this topic and existing literature typically examines adults’ functional status and disengagement or selective investment in leisure activities (Kleiber et al., 2008) rather than examining boredom more broadly. Thus, the purpose of this study was to examine the concept of boredom holistically in the context of leisure among community-dwelling older adults. The study used a multi-method approach utilizing focus groups and projective techniques of sentence completions and drawings. Forty-two older adults were recruited from senior centers and senior communities located in four western US cities. Thematic analysis with a six-step process outlined by Braun and Clarke (2006) was employed to analyze the data. Boredom was described as a personal state (e.g., negative self-perception, low energy and sensation/arousal, lacking motivation) and as activity-related emotions derived from unsatisfied/disengaged leisure participation. Participants also highlighted how life events and circumstances shaped their identity and contributed to boredom. Findings offered meaningful insights into how participants defined boredom, articulated causes of boredom, and strategies they use to alleviate boredom.

